# Clinical trajectories and biomarkers for weight variability in early Parkinson’s disease

**DOI:** 10.1038/s41531-022-00362-3

**Published:** 2022-08-02

**Authors:** Daniele Urso, Daniel J. van Wamelen, Lucia Batzu, Valentina Leta, Juliet Staunton, José A. Pineda-Pardo, Giancarlo Logroscino, Jagdish Sharma, K. Ray Chaudhuri

**Affiliations:** 1 King’s College London, Department of Neurosciences, Institute of Psychiatry, Psychology & Neuroscience, London, UK; 2 Parkinson’s Foundation Centre of Excellence, King’s College Hospital, London, UK; 3 Center for Neurodegenerative Diseases and the Aging Brain, Department of Clinical Research in Neurology, University of Bari ‘Aldo Moro’, “Pia Fondazione Cardinale G. Panico”, Tricase, Lecce, Italy; 4 Radboud University Medical Center, Donders Institute for Brain, Cognition and Behaviour, Department of Neurology, Nijmegen, the Netherlands; 5 HM CINAC. Centro Integral de Neurociencias AC. HM Hospitales. Fundación de Investigación HM Hospitales. HM Hospitales, Madrid, Spain; 6 Centro de Investigación Biomédica en Red de Enfermedades Neurodegenerativas Instituto Carlos III, Madrid, Spain; 7 Geriatric Medicine (Movement Disorders), Lincoln County Hospital, Lincoln, United Kingdom, University of Lincoln, Lincoln, UK

**Keywords:** Parkinson's disease, Prognostic markers

## Abstract

Unexplained weight changes that occur in Parkinson’s disease (PD), are often neglected and remain a poorly understood non-motor feature in patients with PD. A specific ‘Park-weight’ phenotype with low body weight has been described, and our aim was to evaluate the clinical and prognostic trajectories and biomarkers of weight variability in PD. We evaluated body weight-related biomarkers in 405 de novo PD patients and 187 healthy controls (HC) over a 5-year follow-up period from the PPMI database. Body-weight variability was defined as intra-individual variability in body weight between visits. PD patients were categorized as weight losers, gainers, or patients with stable weight. The differential progression of motor and non-motor clinical variables between groups was explored using linear mixed-effects models. Finally, we estimated longitudinal changes in weight as a function of baseline and longitudinal striatal presynaptic dopaminergic transporter imaging. PD patients presented a greater weight variability compared to HC (*p* = 0.003). Patients who developed weight loss had lower CSF amyloid-beta 1–42 (*p* = 0.009) at baseline. In addition, patients with weight loss showed a faster cognitive decline (*p* = 0.001), whereas patients with weight gain showed a slower motor progression (*p* = 0.001), compared to patients with stable weight. Baseline right striatal denervation was a predictor of weight variability in both PD patients and HC (*p* < 0.001). Similarly, weight variability in PD patients was associated with the progression of right striatal denervation (*p* < 0.001). Weight variability and specifically weight loss are more frequent in PD compared to HC, and are associated with specific motor, non-motor and cognitive progression patterns. A greater CSF amyloid burden was present at baseline in patients with subsequent weight loss. Presynaptic dopaminergic imaging in the right striatum may serve as a predictor of future weight changes in PD and HC.

## Introduction

Weight variability, which can be pathological, is a relatively common clinical finding among patients with Parkinson’s disease (PD) and yet remains poorly researched^1^. Weight loss has been reported across all stages of PD^1^ and it has been proposed that it might be a prodromal feature of PD^2–4^. Weight loss has been associated with female sex^5^, levodopa daily dose^6^, dysautonomia^7^, and olfactory dysfunction^8^ as well as greater severity of motor features and dyskinesias^9^, more frequent occurrence of cognitive impairment, and increased disability and mortality in PD patients^10–12^. On the other hand, a considerable number of PD patients show weight gain associated with comorbidities^13^, chronic use of dopamine replacement therapy with associated binge-eating behaviour^14^, and ablative^15^ or functional neurosurgery^16^. Of note, contrasting data are available on the role of body weight as a risk factor for the development of PD^2,3,17,18^.

Although body weight is regulated by many variables including genetic, epigenetic, metabolic, and environmental factors, under physiological conditions, homoeostatic behavioural adaptations tend to preserve a stable body weight^19^. Weight fluctuations may involve changes in energy expenditure, perturbation of homoeostatic control, and eating behaviour modulated by the dopaminergic system^1^, which is known to be altered in PD. Ghrelin and leptin are peptides regarded as modulators of human energy balance, and lower plasma levels of the latter have been identified in patients with PD and weight loss^20,21^. In addition, a variety of additional factors might lead to reduced caloric intake and subsequent weight loss in PD including decreased appetite (due to depression as well as hyposmia), dysphagia, and gastrointestinal dysmotility with altered intestinal absorption. Interestingly, data from preclinical models of PD seems to suggest a possible role of central noradrenergic neurotransmission in weight variations in PD^22^.

Even though the occurrence of unintended weight changes in PD patients has long been recognized^23^, its underlying mechanisms need to be further elucidated. The lack of a clear pathophysiological understanding is accompanied by the scarcity of biomarkers for this complex clinical condition. In healthy elderly individuals, weight loss has been associated with increased PET amyloid uptake^24^, and lower body mass index (BMI) coinciding with decreased levels of amyloid-beta 1–42 (Aβ_1–42_) in cerebrospinal fluid (CSF)^25^, suggesting a higher amyloid burden in these individuals. However, the relationship between weight variability and CSF biomarkers in PD is still unknown.

Furthermore, since the dorsal striatum, which comprises of the putamen and caudate, has been implicated in maintaining caloric requirements^26^, there have been attempts to assess its relationship with weight variability. A recent study found that the putamen-to-caudate DAT ratio at the time of diagnosis predicted subsequent weight change in PD, but only in male patients^27^. In another recent study conducted on healthy individuals, it has been shown that body weight is linked to a higher dopamine receptor availability in the right putamen compared to the left^28^. This relationship in PD, however, remains unexplored. Finally, weight changes have also been associated with urate levels in men with a high cardiovascular risk profile, however, their associations have not been demonstrated in PD^29^. Interestingly, urate levels have been negatively associated with non-motor symptom burden in PD patients^30^.

In this study, we sought to characterize weight variability, their related clinical (motor and non-motor) trajectories, and associated clinical and imaging biomarkers in a large cohort of de novo patients with PD, with a 5-year follow-up with the above-cited possible biomarkers. Specifically, we aimed to explore whether weight variability might be associated with different clinical progression patterns, CSF Aβ_1–42_, urate levels and dopaminergic denervation in the striatum.

## Results

### Weight variability in Parkinson's Disease patients and healthy controls

Demographic and clinical baseline data are shown in Table 1. No demographic differences were found between the two groups. While BMI was not different between PD patients and HC, weight variability (as measured by average successive variability) was higher in patients with PD (1.38 ± 1.5 kg vs 1.08 ± 1.5 kg, *p* = 0.003).

**Table 1:** Baseline Characteristics of Parkinson's Disease patients and healthy controls.

Variables	Healthy controls (*n* = 187)	Parkinson’s disease patients (*n* = 405)	*p*
Age, years	61.1 ± 11.1	61.5 ± 9.7	0.865
Sex, male (%)	65.4%	64.7%	0.863
Education, years	16.1 ± 2.9	15.5 ± 2.9	0.350
MDS-UPDRS I	2.9 ± 2.9	5.5 ± 4.0	–
MDS-UPDRS II	0.4 ± 1.0	5.8 ± 4.1	–
MDS-UPDRS III	1.2 ± 2.2	20.8 ± 8.8	–
MDS-UPDRS total	4.6 ± 4.4	32.1 ± 13.1	–
BMI (Kg/m^2^)	26.8 ± 4.4	27.1 ± 4.6	0.564
Weight variability^a^	1.08 ± 1.5	1.38 ± 1.5	**0.003**
Mean Putamen, SBR	2.14 ± 0.54	0.81 ± 0.28	–
Mean Caudate, SBR	2.97 ± 0.62	1.98 ± 0.55	–

Data are presented as number (%) or mean ± standard deviation.

^a^Weight variability calculated as average successive variability (ASV).

*MDS-UPDRS*Movement Disorders Society Unified Parkinson Disease Rating Scale. *SBR* striatal binding ratio.

### Baseline differences between patients with weight loss, gain and stable weight

Patients with weight gain over the follow-up period of five years were younger at baseline compared to patients with stable weight (*p* = 0.033, Table 2). No other demographic differences were found. Patients with weight loss had more difficulties with their activities of daily living (ADL) (*p* = 0.003) and had lower CSF levels of Aβ_1–42_ (*p* = 0.009) at baseline compared to patients with a stable weight. Other CSF and imaging biomarkers were not different between the three groups (Table 2).

**Table 2:** Baseline differences of de novo Parkinson’s disease patients with subsequent stable weight, weight loss or weight gain after five years of follow-up.

	Stable weight (*n* = 203)	Weight loss (*n* = 134)	Weight gain (*n* = 68)	*P* stable vs loss	*P* stable vs gain
Age, years	62.0 ± 9.9	62.0 ± 9.7	59.4 ± 9.5	0.990	0.033
Sex, male (%)	65.5%	67.9%	60.3%	0.649	0.437
Education, years	15.6 ± 2.8	15.6 ± 3.1	15.3 ± 2.9	0.560	0.481
Binge eating^a^	7.4%	9.7%	11.8%	0.460	0.268
ADL	93.9 ± 5.3	91.9 ± 6.1	93.3 ± 6.1	0.003*	0.588
HY	1.5 ± 0.5	1.6 ± 0.5	1.5 ± 0.5	0.217	0.885
MDS-UPDRS I	5.4 ± 4.0	5.7 ± 3.9	5.3 ± 4.4	0.440	0.589
MDS-UPDRS II	5.4 ± 4.1	6.2 ± 4.3	6.3 ± 4.1	0.057	0.076
MDS-UPDRS III	19.4 ± 8.1	22.0 ± 9.2	22.7 ± 9.5	0.020	0.012
MDS-UPDRS total	30.2 ± 12.5	33.9 ± 13.1	34.3 ± 14.5	0.015	0.083
MoCA	26.9 ± 2.4	27.3 ± 2.2	27.2 ± 2.2	0.028	0.504
UPSIT	22.0 ± 8.0	22.8 ± 8.4	22.2 ± 8.1	0.501	0.759
GDS	2.1 ± 2.2	2.3 ± 2.3	2.8 ± 3.1	0.546	0.130
STAI	63.9 ± 18.0	64.9 ± 17.4	69.8 ± 20.3	0.559	0.033
SCOPA-AUT	8.9 ± 5.7	10.3 ± 6.6	8.9 ± 5.4	0.096	0.981
ESS	5.5 ± 3.1	6.4 ± 3.8	4.8 ± 2.8	0.066	0.130
Weight variability^b^	0.00 ± 0.5	-2.4 ± 1.6	2.2 ± 1.9	**–**	**–**
Mean putamen, SBR	0.84 ± 0.29	0.77 ± 0.25	0.84 ± 0.34	0.038	0.596
Mean caudate, SBR	2.03 ± 0.55	1.91 ± 0.54	2.03 ± 0.57	0.015	0.338
CSF amyloid beta, pg/mL	944.9 ± 389.7	845.1 ± 380.5	936.5 ± 531.0	0.009*	0.356
CSF alpha synuclein, pg/mL	1556.1 ± 671.3	1407.0 ± 543.7	1550.9 ± 853.2	0.942	0.338
CSF tau, pg/mL	169.8 ± 53.4	167.2 ± 52.3	167.3 ± 68.8	0.647	0.928
Serum urate, µmol/L	313.5 ± 77.8	329.1 ± 83.8	307.3 ± 67.7	0.127	0.546

Data are *n* (%) and the mean ± standard deviation.

^a^Item of the Questionnaire for Impulsive-Compulsive Disorders.

^b^Weight variability calculated as average successive variability taking directionality into account.

*HY* Hoehn and Yahr scale, *ADL* Activities of Daily Living, *MDS-UPDRS* Movement Disorders Society Unified Parkinson Disease Rating Scale, *MoCA* Montreal Cognitive Assessment, *SCOPA-AUT* SCOPA-Autonomic, *UPSIT* University of Pennsylvania Smell Identification Test, *GDS* Geriatric Depression Scale, *STAI* State-Trait Anxiety Inventory, *ESS* Epworth Sleepiness Scale, *SBR* striatal binding ratio, *CSF* cerebrospinal fluid.

*Significant *p*-values after Bonferroni correction for multiple testing.

### Clinical trajectories in patients with stable weight, weight loss and weight gain

The longitudinal clinical trajectories of the subgroups are shown in Table 3. Compared to patients with a stable weight, patients with weight loss showed faster progression in MDS-UPDRS II (*p* < 0.001), total MDS-UPDRS (*p* < 0.001), ADL (*p* = 0.001) and MoCA (*p* < 0.001) scores, whereas patients with weight gain showed slower progression in MDS-UPDRS III (*p* < 0.001), and anxiety (STAI, *p* = 0.004) scores.

**Table 3:** Generalized linear mixed analysis for the comparison of the progression over time of clinical variables between de novo Parkinson’s patients with stable weight, weight loss, or weight gain.

Outcome	Baseline (*n* = 406)	Year 1 (*n* = 363)	Year 2 (*n* = 363)	Year 3 (*n* = 359)	Year 4 (*n* = 341)	Year 5 (*n* = 314)	Group × Time effect	
							Est (SE)	*p*
MDS- UPDRS I								
Stable weight	5.4 ± 4.0	6.7 ± 4.7	7.3 ± 4.8	7.8 ± 5.3	8.7 ± 5.7	8.9 ± 5.8		
Weight loss	5.7 ± 3.9	7.3 ± 4.7	7.8 ± 5.5	9.1 ± 5.5	9.9 ± 6.7	10.0 ± 7.1	0.22 (0.09)	0.013
Weight gain	5.9 ± 4.4	6.3 ± 4.3	8.0 ± 5.2	8.2 ± 5.7	8.9 ± 4.6	9.7 ± 5.7	0.25 (0.11)	0.026
MDS- UPDRS II								
Stable weight	5.5 ± 4.1	7.0 ± 4.9	7.5 ± 5.2	8.1 ± 5.4	9.4 ± 6.6	9.4 ± 5.8		
Weight loss	6.2 ± 4.3	8.4 ± 5.2	8.9 ± 5.5	10.3 ± 6.2	10.8 ± 7.2	11.5 ± 7.7	0.32 (0.09)	<0.001*
Weight gain	6.3 ± 4.1	7.2 ± 4.4	7.4 ± 4.5	8.0 ± 4.6	9.4 ± 5.8	10.0 ± 5.7	−0.01 (0.1)	0.945
MDS- UPDRS III								
Stable weight	19.4 ± 8.2	22.7 ± 9.4	25.2 ± 11.5	27.6 ± 12.3	26.9 ± 11.4	30.6 ± 12.4		
Weight loss	22.0 ± 9.2	27.4 ± 10.7	30.1 ± 10.5	33.1 ± 11.4	34.3 ± 12.4	33.7 ± 12.9	0.45 (0.2)	0.854
Weight gain	22.7 ± 9.5	23.7 ± 12.0	27.3 ± 12.8	25.7 ± 11.2	29.5 ± 14.0	29.1 ± 11.8	−0.81 (0.3)	<0.001*
MDS- UPDRS IV								
Stable weight	NA	0.4 ± 1.3	0.7 ± 1.7	1.0 ± 1.8	1.6 ± 2.5	2.1 ± 2.8		
Weight loss	NA	0.4 ± 1.3	0.9 ± 2.1	1.0 ± 2.2	1.6 ± 2.9	2.0 ± 3.2	−0.003 (0.02)	0.962
Weight gain	NA	0.6 ± 1.6	0.3 ± 0.9	0.5 ± 1.3	1.3 ± 2.4	2.4 ± 2.8	0.02 (0.09)	0.696
MDS-UPDRS								
Stable weight	30.3 ± 12.5	36.1 ± 14.5	39.4 ± 17.4	42.6 ± 18.1	46.3 ± 17.9	48.0 ± 18.5		
Weight loss	33.9 ± 13.1	43.6 ± 16.8	46.9 ± 16.6	52.7 ± 18.8	53.8 ± 22.1	53.8 ± 21.2	1.07 (0.32)	0.001*
Weight gain	34.3 ± 14.5	37.4 ± 16.4	42.8 ± 16.6	41.5 ± 16.6	46.8 ± 20.1	50.0 ± 18.9	−0.40 (0.41)	0.340
ADL								
Stable weight	94.0 ± 5.4	91.0 ± 7.0	89.2 ± 8.3	88.4 ± 7.8	86.6 ± 10.3	86.0 ± 10.4		
Weight loss	91.9 ± 6.2	89.7 ± 6.3	87.4 ± 8.2	86.0 ± 8.5	83.0 ± 10.3	80.5 ± 16.4	0.07 (0.18)	<0.001*
Weight gain	93.4 ± 6.2	91.2 ± 6.3	90.6 ± 6.4	89.5 ± 7.5	88.2 ± 3.7	86.9 ± 8.0	0.27 (0.23)	0.237
MoCA								
Stable weight	26.9 ± 2.4	26.4 ± 2.7	26.3 ± 2.9	26.6 ± 2.8	26.8 ± 3.1	27.0 ± 2.9		
Weight loss	27.3 ± 2.2	26.3 ± 3.0	26.2 ± 3.6	26.1 ± 3.3	25.7 ± 4.1	25.4 ± 4.6	0.35 (0.05)	<0.001^*^
Weight gain	27.2 ± 2.2	26.1 ± 2.7	26.2 ± 3.2	26.5 ± 3.1	26.5 ± 3.7	27.2 ± 2.7	0.05 (0.06)	0.448
GDS								
Stable weight	2.1 ± 2.2	2.3 ± 2.8	2.3 ± 2.6	2.3 ± 2.6	2.5 ± 2.6	2.5 ± 2.6		
Weight loss	2.3 ± 2.3	2.7 ± 3.1	2.7 ± 2.9	3.1 ± 3.1	2.8 ± 3.1	3.3 ± 3.2	0.10 (0.05)	0.061
Weight gain	2.8 ± 3.1	2.7 ± 2.7	3.4 ± 3.4	2.7 ± 2.7	2.6 ± 2.8	2.7 ± 2.6	−0.10 (0.06)	0.116
STAI								
Stable weight	63.9 ± 18.0	64.1 ± 18.5	62.9 ± 17.4	63.6 ± 17.9	64.2 ± 18.1	63.7 ± 18.1		
Weight loss	64.7 ± 17.3	65.7 ± 18.3	67.3 ± 20.1	66.7 ± 20.7	65.7 ± 19.6	67.8 ± 22.7	0.42 (0.30)	0.167
Weight gain	69.7 ± 20.3	68.0 ± 19.5	68.1 ± 19.0	65.3 ± 18.5	65.6 ± 19.1	63.4 ± 16.5	−1.10 (0.39)	0.004*
SCOPA								
Stable weight	8.9 ± 5.7	10.5 ± 6.2	10.6 ± 6.3	11.3 ± 6.6	11.8 ± 7.1	12.7 ± 8.1		
Weight loss	10.2 ± 6.6	12.3 ± 6.8	12.7 ± 7.0	14.2 ± 7.8	14.2 ± 8.2	15.2 ± 9.0	0.20 (0.10)	0.061
Weight gain	8.8 ± 5.4	9.83 ± 6.0	11.2 ± 5.3	11.8 ± 6.2	12.9 ± 6.0	13.6 ± 5.5	0.18 (0.13)	0.168
ESS								
Stable weight	5.5 ± 3.1	6.1 ± 4.1	6.7 ± 4.4	7.2 ± 4.4	7.4 ± 4.5	7.6 ± 4.5		
Weight loss	6.4 ± 3.8	6.5 ± 4.0	6.6 ± 4.0	7.6 ± 4.7	7.6 ± 4.8	8.0 ± 4.7	−0.06 (0.08)	0.433
Weight gain	4.8 ± 2.8	5.6 ± 3.5	6.8 ± 4.0	7.0 ± 4.0	7.3 ± 4.8	7.7 ± 5.2	0.18 (0.09)	0.060

*NA* not applicable, *MDS-UPDRS* Movement Disorders Society Unified Parkinson Disease Rating Scale, *MoCA* Montreal Cognitive Assessment. *SCOPA-AUT* SCOPA-autonomic, *UPSIT* University of Pennsylvania Smell Identification Test, *GDS* Geriatric Depression Scale, *STAI* State-Trait Anxiety Inventory, *ESS* Epworth Sleepiness Scale.

*Significant *p*-values after Bonferroni correction for multiple testing.

### Binge eating disorder and dopaminergic medications

Binge eating disorder was more prevalent in weight gainers (Est: 0.35, SE: 0.15, *p* = 0.02) compared to those with a stable weight, although LEDD or the use of DAs was not different between the groups. However, in the whole sample, the clinical trajectory of binge eating disorder was significantly associated with the use of DAs (Est: 0.59, SE: 0.16, *p* < 0.001), with a trend towards statistical significance for LEDD (*p* = 0.051).

### Urate and weight

Serum urate leves were associated with BMI at baseline (*r* = 0.398, *p* < 0.001). Furthermore, compared to patients with a stable weight, patients with weight gain showed increasing levels of serum urate (*p* < 0.001, Supplementary Table 1, Supplementary Fig. 1), while patients with weight loss demonstrated decreasing serum urate levels (*p* < 0.001).

### Weight variability and striatal dopaminergic integrity in Parkinson's Disease patients and healthy controls

We found a significant association between weight variability and right striatal DaT binding ratios (*p* < 0.001, Table 4), but not left striatum binding ratios. We also explored whether dopaminergic denervation at baseline could predict weight variability. Mean, as well as right striatum, putamen, and caudate DaT binding ratios were all predictive of weight variability (*p* < 0.001, Table 5), while the left-sided regions were not. Finally, we explored the directionality of this relationship in the different subgroups of PD patients (Supplementary Table 2). We found that in the group of patients with weight loss, weight variability was predicted by mean striatal (*p* < 0.005), right putamen (*p* < 0.001), left caudate (*p* = 0.003), and right striatal (*p* = 0.002) DaT binding ratios. In the group of patients with weight gain, weight was predicted by mean striatum (*p* = 0.001), putamen (*p* < 0.001), right putamen (*p* < 0.001) caudate (*p* < 0.001), and striatum (*p* < 0.001) DaT binding ratios. Finally, we found that in HC also, right putamen binding ratios (*p* < 0.001) were predictive of weight variability (Table 5).

**Table 4:** Longitudinal changes of weight in Parkinson’s patients are associated with longitudinal dopaminergic imaging.

Variable × time effect	Est (SE)	*P*
Mean putamen	0.74 (0.32)	0.020
Mean caudate	0.28 (0.14)	0.041
Mean striatum	0.46 (0.20)	0.023
Left putamen	0.43 (0.29)	0.142
Right putamen	0.71 (0.27)	0.009
Left caudate	0.16 (0.13)	0.223
Right caudate	0.34 (0.13)	0.008
Left striatum	0.13(0.09)	0.151
Right striatum	0.25 (0.09)	0.001*

Main and interaction effects of the linear mixed-effects models estimating the longitudinal changes of weight in PD patients as function of longitudinal presynaptic dopaminergic transporter imaging. The model was controlled for age, sex and disease duration.

*Significant *p*-values after Bonferroni correction for multiple testing.

**Table 5:** Longitudinal changes of weight are predicted by baseline dopaminergic imaging in Parkinson’s patients and healthy controls (HC).

Variable × time effect	PD		HC	
	Est (SE)	*P*	Est (SE)	*P*
Mean putamen	1.00 (0.18)	<0.001*	0.30 (0.11)	0.009
Mean caudate	0.34 (0.09)	<0.001*	0.08 (0.10)	0.421
Mean striatum	0.59 (0.13)	<0.001*	0.189 (0.11)	0.093
Left putamen	0.25 (0.15)	0.109	0.25 (0.11)	0.026
Right putamen	1.06 (0.15)	<0.001*	0.31(0.11)	0.004*
Left caudate	0.16 (0.08)	0.068	0.13(0.97)	0.173
Right caudate	0.41 (0.08)	<0.001*	0.01(0.09)	0.858
Left striatum	0.11 (0.06)	0.063	0.10 (0.05)	0.060
Right striatum	0.35 (0.05)	<0.001*	0.08 (0.05)	0.132

Main and interaction effects of the linear mixed-effects models estimating the longitudinal changes of weight in PD patients and healthy controls as function of baseline presynaptic dopaminergic transporter imaging, while controlling for age, sex and disease duration (only in the PD group).

*Significant *p*-values after Bonferroni correction for multiple testing.

## Discussion

In this longitudinal study we have demonstrated that (1) pathological weight loss may be associated with lower baseline levels of CSF Aβ_1-42_ in patients with early and de novo PD, (2) both weight loss and weight gain are associated with right striatal dopaminergic denervation in PD patients and HC, (3) there is a relationship between weight variability and serum urate levels, and (4) motor and non-motor longitudinal clinical trajectories of PD patients with either weight gain or weight loss are different.

Unintended weight changes and weight loss have long been recognized in PD patients. In the pre-levodopa era, PD was a disease associated with malnutrition and obesity was rarely observed, even up to a few decades ago^31^. More recently, in optimally treated patients, PD subjects can be overweight or even obese^32^, and this change of phenotype is probably related to modern pharmacotherapy (most likely the use of DAs), but may also be at least partly due to the overall increase of obesity in modern society^1^. However, in PD, weight changes, and especially weight loss, can become pathological and intrusive, and be prevalent from the prodromal to the advanced stage^1^. As such, this feature of PD needs a clearer research focus, as the pathophysiological basis remains an unmet need. Recent work has attempted a “deep dive” into the clinical associates of abnormal weight loss in PD, and weight loss appears to be associated with a more rapid decline in motor function, cognitive impairment, and disability and mortality in general. Cummings et al.^33^ recently showed that weight loss occurring within one year after PD diagnosis was independently associated with an increased risk of dependency, dementia and death. In another study from the NINDS Exploratory Trials, Wills and colleagues found that the weight loss group’s mean motor UPDRS score increased by 1.48 points per visit when compared to the weight-stable group’s mean motor UPDRS score^11^.

Currently, there is a scarcity of available biomarkers of weight variability in PD. In our cohort, de novo patients with subsequent weight loss had lower levels of CSF Aβ_1–42_ compared to patients with a stable weight. This association is in line with the finding that patients with weight loss have a faster progression in relation to cognitive dysfunction^33^, which has been linked with CSF Aβ_1–42_^34^. The presence of a greater amyloid burden has been previously demonstrated in healthy elderly individuals and individuals with mild cognitive impairment (MCI), where weight was associated with increased PET amyloid uptake^24^. Another study found a relationship between lower BMI and decreased levels of amyloid in CSF in cognitively normal and MCI individuals^25^. Furthermore, weight loss has been described in other neurodegenerative conditions, especially dementia^35–37^. Thus, amyloid burden, or protein misfolding in general, could explain both this commonality in neurodegeneration and a more rapid clinical progression, and suggests that weight loss is a condition that could be partially related to an underlying amyloid pathology load, already present during the first stages of clinically manifested PD^38^.

We found that both weight loss and weight gain are associated with right striatal dopaminergic denervation in PD patients and HC. While the dopaminergic ventral striatum has been involved in the reward system behind food intake in Binge Eating Disorder, dopamine in the dorsal striatum has been implicated in maintaining caloric requirements for survival and in “non-hedonic” food motivation^1,26^. Recent studies have demonstrated longitudinal associations of weight change with striatal dopaminergic degeneration. DaT binding at baseline seemed to predict weight changes, with putamen-to-caudate nucleus ratio as an independent predictor for weight change, although only in male subjects^27,39^. Whether right-left asymmetry in striatal binding ratios contributes to weight variability, as it has been shown in our study and one previous study^28^, remains to be further explored.

Urate, the soluble form of uric acid, is an important physiological antioxidant able to scavenge free oxygen radicals and interact with other antioxidant systems^40,41^. Increasing epidemiological and clinical evidence have supported the view that higher urate levels could be associated with a decreased risk of PD and a slower disease progression^40^. We found that weight variability was associated with urate level, since it increases in patients with weight gain and decreases in patients with weight loss. Interestingly, a recent observational study observed that urate levels were negatively associated with global NMS burden in PD patients, with a specific link to the miscellaneous domain of the NMS scale, which included weight variability^30^. A possible explanation for weight variability in PD could, therefore, be that these changes are mediated through urate. In this respect, it is interesting to note that in rats high urate diet is associated with the expression of pro-inflammatory cytokines and increased gliosis in the hypothalamus, especially in the mediobasal hypothalamus^42^, containing, for example, the infundibular and ventromedial nuclei involved in feeding and neuroendocrine control^43^.

Non-motor endophenotyping of PD is a recent, albeit controversial, concept of great clinical focus, and may aid subtype-specific medicine^44,45^. Weight variability is an essential constituent of the recently described “circle of personalised medicine”^46^. Weight loss is also the underpinning anchor in the proposed ‘Park-weight’ PD phenotype^9^. These patients have been shown to be affected by severe loss of olfaction, a symptom that could increase their risk of unexplained weight loss, developing dyskinesia, and worse disease prognosis^9^. However, the full range of symptoms associated with this phenotype has not yet been extensively explored or defined, and our report attempts to unravel some of the clinical associates of this endophenotype. Firstly, this analysis, with datamining from an independent cohort of de novo PD patients, suggests that a subgroup of patients with PD have more pronounced weight variability compared to HC, confirming the validity of the original description of the park-weight phenotype and nonmotor subtype of PD. We can also confirm the observations of Sharma et al.^9^, suggesting that PD patients with weight loss have a more rapid progression of motor symptoms, cognitive decline, and disability. Interestingly, we found that patients with weight gain had a slower progression of motor function. Therefore, we confirm that weight variability may have a critical clinical significance in PD, with weight loss as a driver of poor outcomes, and weight gain associated with slower motor progression in the long term. We also found that patients with weight loss had more motor disability measured by the Schwab and England Activities of Daily Living (ADL) scale at baseline compared with patients with stable weight, which is in line with a previous study showing an association between difficulty in eating and drinking and weight loss^11^. As previously reported^14^, binge eating disorder, a manifestation of Impulsive Control Disorder, was associated with the use of DAs.

Strengths of the current study include the use of a large sample from an international cohort of patients of early de novo PD and HC with a follow-up of up to 5 years. Contrary to other studies where weight changes have been defined as a change between the baseline and last visits, here we used body-weight variability by calculating the average successive variability, which takes into account intra-individual variability in body weight between each visit^47^. Moreover, we have comprehensively characterized weight variability, confirming that this is more prevalent in PD compared with the HC, describing the motor and non-motor trajectories of patients with weight loss and weight gain. In addition, we included in our analysis both right and left striatal regions to explore possible lateralization of function. Nevertheless, some limitations should be recognised in this study. Data on other potential confounders, including intentional weight change, dietetic interventions, non-dopaminergic medication use, gut motility, potential external stressors, exercise and nutritional status, were unavailable. Secondly, olfactory function was only assessed at baseline preventing us from exploring its longitudinal association with weight variability. Further longitudinal studies using more comprehensive nutritional assessments, including specific clinical scales and anthropometric measurements of body composition, are required to improve the characterization and the identification of biomarkers of weight variability in PD.

In conclusion, in this longitudinal study, we found that weight loss was associated with poor clinical outcomes and with a lower level of CSF Aβ_1–42_ at baseline, while a more favourable progression of motor function was observed in patients with weight gain. Weight variability was associated with urate levels in PD and with right striatal dopaminergic integrity in both PD patients and HC. As such, presynaptic dopaminergic imaging and urate levels may serve as a predictor of weight variability in PD.

## Methods

### Subjects

Data used in the preparation of this article were obtained from the Parkinson Progression Marker Initiative (PPMI)^48^. The PPMI is an ongoing prospective, observational, international, multicentre study aimed at identifying clinical biomarkers of PD in a large cohort of participants with early PD at enrolment alongside healthy controls. The aims and methodology of the study have been extensively published elsewhere and are available at www.ppmi-info.org/study-design. Inclusion criteria for PD patients were age 30 years or older, diagnosis of PD (based on one of the following: the presence of (1) asymmetrical resting tremor or (2) asymmetrical bradykinesia or (3) at least two of either of resting tremor, bradykinesia, and rigidity), and a disease duration of 1–24 months, Hoehn and Yahr (H&Y) stage of 1 to 2, and presence of striatal dopamine transporter deficit on ^123^I-FP-CIT SPECT. The data were collected from more than 33 clinical sites in 11 countries. The PPMI study was approved by the local Institutional Review Boards of all participating sites and written informed consent for imaging data and clinical questionnaires was obtained from each participant at the time of enrolment. All methods were performed in accordance with the relevant guidelines and regulations. We obtained data from the PPMI database on 4 May 2020 in compliance with the PPMI Data Use Agreement.

### Clinical assessment

We included data from 405 de novo PD patients and 187 HC with complete information on weight at baseline and throughout the 5-year follow-up. Follow-up visits were performed annually. Data extracted included demographics, age at onset, disease duration, baseline and longitudinal body weight and height, Hoehn and Yahr (HY) staging, Movement Disorder Society-Unified Parkinson’s Disease Rating Scale (MDS-UPDRS), SCOPA-Autonomic (SCOPA-AUT), Montreal Cognitive Assessment (MoCA), University of Pennsylvania Smell Identification Test (UPSIT), Geriatric Depression Scale (GDS), State-Trait Anxiety Inventory (STAI), Epworth Sleepiness Scale (ESS), The Schwab and England Activities of Daily Living (ADL) scale. Binge eating was evaluated using the Questionnaire for Impulsive-Compulsive Disorders in Parkinson’s Disease (QUIP). We also extracted data on medications including levodopa equivalent daily dosage (LEDD) and the use of dopamine agonist (DA). UPSIT scores were only available at baseline.

### Biomarkers assessment

SPECT images of DAT radioligand binding were acquired at baseline and years 1, 2, and 4 in accordance with PPMI neuroimaging protocols^49^. After pre-processing, regions of interest were placed on the left and right caudate and putamen. Occipital cortex was used as the reference region. Striatal binding ratios (SBRs) were calculated as target ROI binding intensities normalized by the reference region. Biochemical analyses of uric acid have been carried out in Covance laboratories in a uniform fashion, as per the study protocol^50^. Measurements of Aβ1–42, total tau and p-tau were obtained for CSF samples at the University of Pennsylvania using the multiplex Luminex xMAP platform (Luminex Corp: Austin, Texas, USA) with research-use-only Fujirebio-Innogenetics INNO-BIA AlzBio3 immunoassay kit-based reagents (Innogenetics Inc: Harvard, MA, USA)^49^. CSF α-synuclein was analyzed at a central laboratory (Covance, MA, US) using a commercially available enzyme-linked immunosorbent assay kit^51^. This kit was developed and optimized for PPMI.

### Body mass index and weight variability

Weight and height have been measured at baseline and annually. BMI was calculated as weight in kilograms divided by height in square metres. Weight variability was calculated by the average successive variability (ASV) method^47^. In detail, weight variability was determined by calculating the averaged absolute values of the differences in weight between visits. PD patients were then stratified according to the median value of ASV into patients with Stable Weight (below the median ASV) or Unstable Weight (above the median ASV)^47^. Unstable Weight patients were further divided according to the directionality of AVS into the weight loss group (negative ASV) and weight gain group (positive ASV).

### Statistical analysis

Between-group comparisons were performed by one-way ANOVA or Mann–Whitney *U* test for normally or non-normally distributed variables, respectively. Categorical variables were compared using Pearson Chi-square. Correlations were performed using the Pearson correlation coefficient test. The differential progression of clinical variables between groups was calculated using linear mixed effects (LME) or mixed effects logistic regression methods. Finally, LME models estimated the longitudinal changes in weight as a function of baseline or longitudinal presynaptic dopaminergic transporter imaging. All models were controlled for age, sex, disease duration and LEDD. Values of *p* < 0.05 were considered as statistically significant, and Bonferroni post-hoc correction was used for multiple comparisons.

## Supplementary information

Supplementary Material.docx

Supplementary Figure1.jpg

## Data Availability

All data used in this study are available from the PPMI database (www.ppmi-info.org/data).
