# Supplementary material for: Clinical trajectories and biomarkers for weight variability in early Parkinson’s disease
Source: NPJ Parkinsons Dis. 2022 Aug 2;8:95. doi: 10.1038/s41531-022-00362-3 (PMC9345874; doi:10.1038/s41531-022-00362-3)
Supplement: Supplementary file 1 — Supplementary Material.docx [file 41531_2022_362_MOESM1_ESM.docx]

**Supplementary Material

Clinical trajectories and biomarkers for weight variability in early Parkinson’s disease.**

Daniele Urso*^1,2,3^, Daniel J van Wamelen^1,2,4^, Lucia Batzu^1,2^, Valentina Leta^1,2^, Juliet Staunton^1,2^, José A Pineda-Pardo^5,6^, Giancarlo Logroscino^3^, Jagdish Sharma^7^, K Ray Chaudhuri^1,2^

1. King’s College London, Department of Neurosciences, Institute of Psychiatry, Psychology & Neuroscience, United Kingdom

2. Parkinson’s Foundation Centre of Excellence, King's College Hospital, Denmark Hill, London

3. Center for Neurodegenerative Diseases and the Aging Brain, Department of Clinical Research in Neurology, University of Bari 'Aldo Moro', "Pia Fondazione Cardinale G. Panico", Tricase, Lecce, Italy

4. Radboud University Medical Center, Donders Institute for Brain, Cognition and Behaviour, Department of Neurology, Nijmegen, the Netherlands

5. HM CINAC. Centro Integral de Neurociencias AC. HM Hospitales. Fundación de Investigación HM Hospitales. HM Hospitales. Madrid, Spain

6 - Centro de Investigación Biomédica en Red de Enfermedades Neurodegenerativas Instituto Carlos III, Madrid, Spain

7- Geriatric Medicine (Movement Disorders), Lincoln County Hospital, Lincoln, United Kingdom; University of Lincoln, Lincoln, United Kingdom

**Corresponding author**

Daniele Urso, King’s College London, Department of Basic and Clinical Neurosciences, Institute of Psychiatry, Psychology & Neuroscience, De Crespigny Park, London, SE5 8AF, United Kingdom

Email: daniele.urso@kcl.ac.uk

**Supplementary Table 1.** Generalized Linear Mixed Analysis for the comparison of the progression of serum urate level between Parkinson’s patients with stable weight, weight loss and weight gain.

| Outcome | Baseline | Year 1 | Year 2 | Year 3 | Year 4 | Year 5 | Group x Time Effect | |
| --- | --- | --- | --- | --- | --- | --- | --- | --- |
|  |  |  |  |  |  |  | Est (SE) | p |
| Urate (Serum)  Stable Weight  Weight Loss  Weight Gain | 313.5±77.8 329.1±83.8 307.3±67.7 | 313.1±74.8 311.0±78.0 308.4±73.5 | 312.4±76.8 297.0±72.1 315.9±76.1 | 319.3±76.5 299.5±77.5 315.1±79.0 | 316.0±78.8 296.7±68.2 325.8±81.7 | 313.2±78.8 295.3±79.5 331.8±84.6 | -7.27(1.03) 4.77(1.31) | **<0.001* <0.001*** |

**Supplementary Table 2.** Main and interaction effects of the linear mixed-effects models estimating the longitudinal changes of weight in Parkinson’s patients as function of baseline presynaptic dopaminergic transporter imaging, while controlling for age, sex and disease duration in different groups (stable weight, weight loss and weight gain).

| **Variable x time effect** | **Stable weight** | | **Weight Loss** | | **Weight Gain** | |
| --- | --- | --- | --- | --- | --- | --- |
|  | **Est (SE)** | ***P*** | **Est (SE)** | ***P*** | **Est (SE)** | ***P*** |
| Mean Putamen | -0.07(0.17) | 0.679 | 1.06(0.38) | **0.005*** | 2.15 (0.40) | **<0.001*** |
| Mean Caudate | -0.15 (0.08) | 0.076 | 0.51(0.17) | **0.003*** | 0.45 (0.23) | 0.051 |
| Mean Striatum | -0.17 (0.12) | 0.165 | 0.78(0.25) | **0.002*** | 1.01(0.31) | **0.001*** |
| Left Putamen | -0.09 (0.13) | 0.471 | 0.35(0.32) | 0.266 | 1.13 (0.43) | 0.008 |
| Right Putamen | 0.01 (0.14) | 0.935 | 1.05 (0.30) | **<0.001*** | 1.49 (0.27) | **<0.001*** |
| Left Caudate | -0.16(0.07) | 0.034 | 0.48 (0.16) | **0.003*** | 0.09 (0.22) | 0.667 |
| Right Caudate | -0.09 (0.08) | 0.268 | 0.41 (0.15) | 0.009 | 0.65 (0.20) | **<0.001*** |
| Left Striatum | -0.08 (0.0) | 0.089 | 0.29 (0.11) | 0.013 | 0.19 (0.15) | 0.217 |
| Right Striatum | -0.04 (0.05) | 0.471 | 0.34 (0.11) | **0.002*** | 0.54 (0.12) | **<0.001*** |

*Significant p-values after Bonferroni correction for multiple testing.

**Supplementary Figure 1**. Predicted trajectories of the progression of serum urate level between Parkinson’s patients with stable weight, weight loss and weight gain**.**
