# Supplementary figures and images for: Clinical trajectories and biomarkers for weight variability in early Parkinson’s disease
Source: NPJ Parkinsons Dis. 2022 Aug 2;8:95. doi: 10.1038/s41531-022-00362-3 (PMC9345874; doi:10.1038/s41531-022-00362-3)

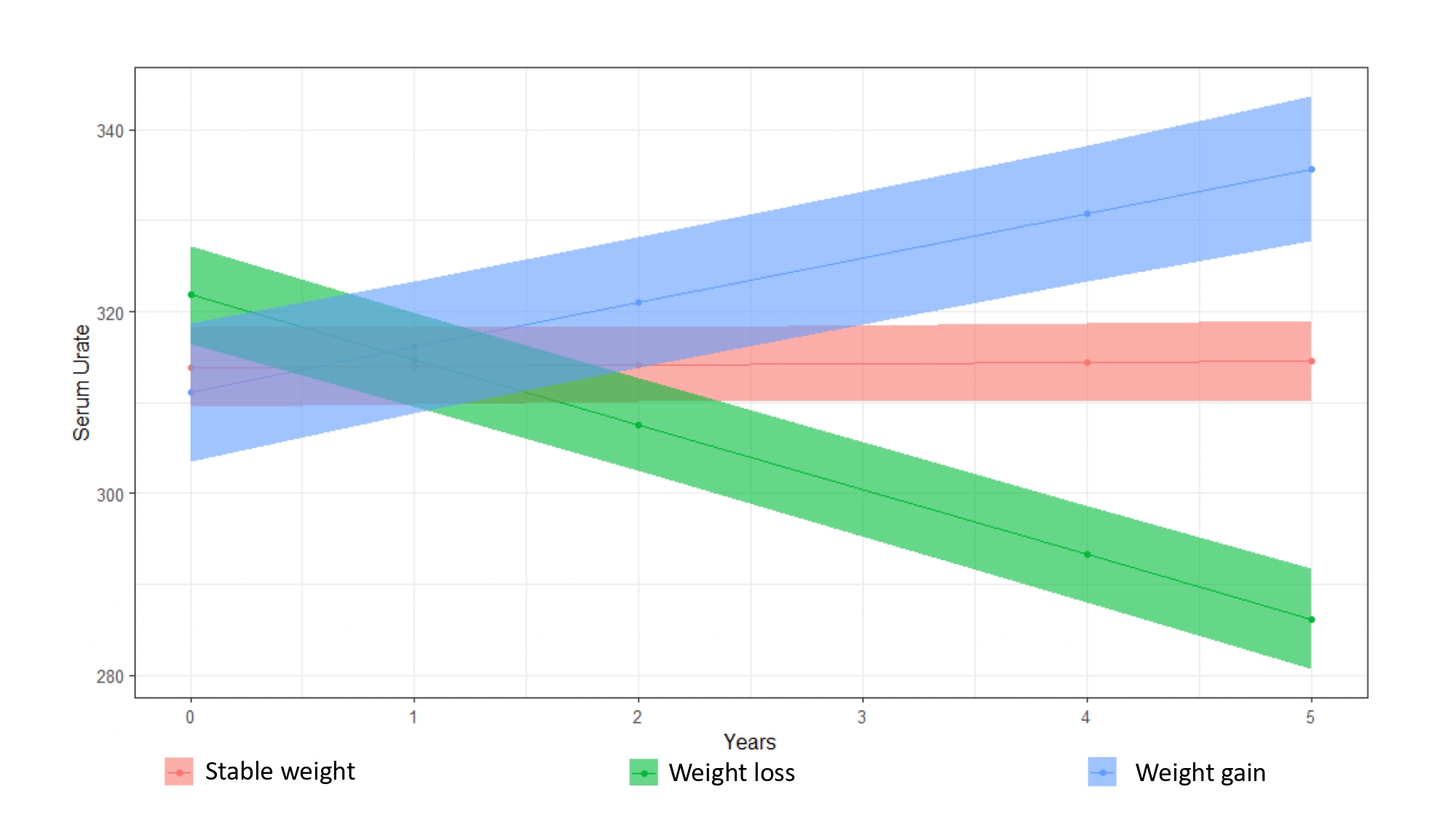

Supplement: Supplementary file 2 — Supplementary Figure1.jpg [file 41531_2022_362_MOESM2_ESM.jpg]
